# The Influence of Electromagnetic Radiation Generated by a Mobile Phone on the Skeletal System of Rats

**DOI:** 10.1155/2015/896019

**Published:** 2015-02-01

**Authors:** Karolina Sieroń-Stołtny, Łukasz Teister, Grzegorz Cieślar, Dominik Sieroń, Zbigniew Śliwinski, Marek Kucharzewski, Aleksander Sieroń

**Affiliations:** ^1^School of Health Sciences in Katowice, Department of Physical Medicine, Chair of Physiotherapy, Medical University of Silesia, Medyków Street 12, 40-752 Katowice, Poland; ^2^Orthopaedics and Motor System Trauma Surgery Unit, Saint Joseph Hospital, Okrzei Street 27, 43-190 Mikołów, Poland; ^3^School of Medicine with the Division of Dentistry in Zabrze, Department and Clinic of Internal Diseases, Angiology and Physical Medicine in Bytom, Medical University of Silesia, Batorego Street 15, 41-902 Bytom, Poland; ^4^Dr. Janusz Daab District Trauma Surgery Hospital, Bytomska Street 62, 41-940 Piekary Śląskie, Poland; ^5^Department of Manual Therapy, Institute of Physiotherapy, Jan Kochanowski University, IX Wieków Kielc Street 19, 25-317 Kielce, Poland; ^6^School of Medicine with the Division of Dentistry in Zabrze, Chair and Department of Descriptive and Topographic Anatomy, Medical University of Silesia, Jordana Street 19, 41-808 Zabrze, Poland

## Abstract

The study was focused on the influence of electromagnetic field generated by mobile phone on the skeletal system of rats, assessed by measuring the macrometric parameters of bones, mechanical properties of long bones, calcium and phosphorus content in bones, and the concentration of osteogenesis (osteocalcin) and bone resorption (NTX, pyridinoline) markers in blood serum. The study was carried out on male rats divided into two groups: experimental group subjected to 28-day cycle of exposures in electromagnetic field of 900 MHz frequency generated by mobile phone and a control, sham-exposed one. The mobile phone-generated electromagnetic field did not influence the macrometric parameters of long bones and L_4_ vertebra, it altered mechanical properties of bones (stress and energy at maximum bending force, stress at fracture), it decreased the content of calcium in long bones and L_4_ vertebra, and it altered the concentration of osteogenesis and bone resorption markers in rats. On the basis of obtained results, it was concluded that electromagnetic field generated by 900 MHz mobile phone does not have a direct impact on macrometric parameters of bones; however, it alters the processes of bone mineralization and the intensity of bone turnover processes and thus influences the mechanical strength of bones.

## 1. Introduction

A substantial part of the human population is exposed to electromagnetic fields generated by mobile phones. Statistics (ITU Yearbook of Statistics 2012: 38th edition; Chronological Time Series 2002–2011, edited by International Telecommunication Union; English Edition online: http://www.itu.int/ITU-D/ict/publications/yb/index.html) show that, in December 2011, the number of mobile network registered users globally amounted to 5.981 billion, including 741 m in Europe and 50.7025 m in Poland. On a global scale, this corresponds to a saturation equivalent to 86.7 phones per 100 residents. Despite the common exposure of the human population to the influence of electromagnetic fields generated by mobile phones and base transceiver stations, there is little documented research devoted to the influence of the field on mammals, including humans.

Mobile phones emit electromagnetic radiation within the microwave frequency range (900–2450 MHz), which may pose a danger to human health due to causing thermal as well as nonthermal effects, such as genotoxicity, carcinogenicity, and sleep disorders [1–3].

Mobile phone-generated radio waves reach powers up to 2 W. The maximum output power of a mobile phone is determined by norms established in each country. The coefficient of the radiation absorbed by the human body is expressed as SAR (specific absorption rate) in Watts per kilogram of body weight (W/kg of body weight). In Europe, including Poland, the recommendations made by the International Commission on Non-Ionising Radiation Protection (1998) and adopted by the European Union Council set a SAR limit of 2.0 W/kg in 10 g of tissue, while, in the United States, all mobile phones must comply with the Federal Communications Commission SAR limit of 1.6 W/kg in 1 g of tissue. The value of SAR for specific mobile phones is stated by the manufacturer.

Biological effects of the influence of low-frequency electromagnetic field on living organisms have been the research topic of much experimental research in recent years. It was demonstrated that electromagnetic fields accelerate the regenerative processes of many types of tissue, including the processes of building up and thickening of bones, shortening the time of postfracture bone union, and the creation of proper union in pseudoarthrosis. In vitro examinations demonstrated a stimulating effect of the low-frequency pulsed electromagnetic field on osteogenesis and intensification of the mRNA expression of the bone morphogenetic proteins (BMP-2 and BMP-*μ*) in rat osteoblast cultures [4–7]. So far, however, it remains debatable whether there exists influence of mobile network generated electromagnetic fields (frequencies of 900 to 2500 MHz) on living organisms. International experimental and epidemiological research which has been carried out for the last ten years has not been able to determine conclusively whether there exists the possibility of causing activity changes in cells and tissue by such fields. The researches investigating biological effects and health consequences of prolonged exposure to mobile phone-generated electromagnetic fields are still being conducted, and research projects such as Mobi-Kids and Kosmos are planned to last for decades [8–11]. Until now, there has been no systematic research as to the influence of mobile network generated electromagnetic fields on the skeletal system of mammals.

The aim of the research carried out in this paper was to assess the influence of the electromagnetic fields generated by Nokia 5110 (frequency of 900 MHz) mobile phone on rat skeletal system. To achieve this aim, it was necessary to determine the effects of the aforementioned field on the bone micrometric parameters, mechanical properties, and mineral content, including calcium and phosphorus. The research also attempted to determine the influence of the field on the processes of bone remodelling by measuring the markers of bone turnover (osteogenesis and bone resorption) as well as calcium and phosphorus concentration in the serum.

## 2. Material and Methods

### 2.1. Animals

The research was undertaken on 10-week-old male rats of Wistar strain taken from the Central Animal Quarters of the Medical University of Silesia in Katowice, with permission of the local bioethical committee (permission number 65/2008), and it has been carried out in accordance with EU Directive 2010/63/EU for animal experiments. During the experiments, the animals had optimal environmental conditions, retaining the 24-hour cycle (12-hour day phase in artificial light and 12-hour night phase). The animals were fed daily with 25 g of laboratory feed (Labofeed-B) (manufactured by Wytwórnia Pasz “Morawski,” Poland), containing 8.70 g of calcium per kg and 7.44 g of phosphorus per kg of granulate. They also had unconstrained access to water. The animals were provided with daily sanitary-hygienic service. They were placed in cages of 10 individuals, in laboratory with stable air temperature (25°C) and humidity (60%). 20 rats randomly divided into two groups of ten were used for the research. The principles of experimental research ethics and the requirements of good laboratory research practice demand using the smallest possible representative groups of animals throughout the research. Obtaining statistically significant results for a small study group raises the importance of the data acquired by this method. The number of animals in both groups was specified according to appropriate statistical methods.

### 2.2. Research Scheme

#### 2.2.1. Exposure of Animals to the Electromagnetic Field Generated by Nokia 5110 Mobile Phone

The research was conducted on two groups of rats.

**Figure 1:**
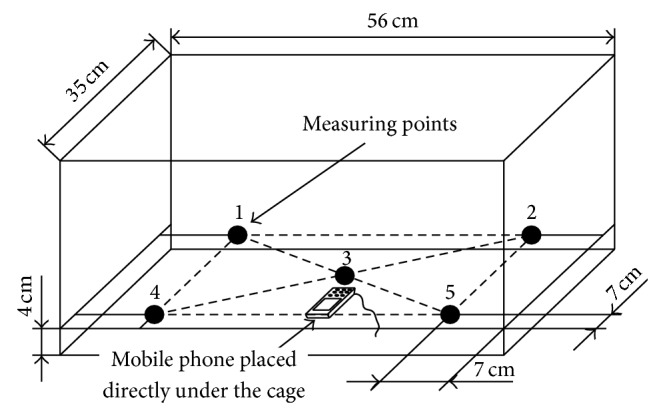
Outline of the exposure workstation with measuring points.

1. The control group (*C*) was subjected to 28-day cycle of sham exposures. While placed in the exposure cage (Figure 1), electromagnetic field was not generated. The time of sham exposure was 22 hours per day with a 2-hour break between 8:00 and 10:00 AM.
2. The experimental group (*E*_*m*_) was subjected to 28-day cycle of exposures to the electromagnetic field generated by a mobile phone (900 MHz frequency Nokia 5110). For this phone, the highest SAR value under the ICNIRP guidelines for use of the device (tests conducted using standard operating positions with the device transmitting at its highest certified power level in all tested frequency bands) stated by the manufacturer was 0.69 W/kg of body weight. As the SAR value depends among others on how close the phone is placed to the body, in this experimental model in which the animals were allowed to move freely inside of a cage during the exposure, the measurement of precise real value of SAR for animal moving inside exposure workstation was not possible. That is, the strength of the electromagnetic field generated by mobile phone was measured as the average density of the electromagnetic field.

The experiment was conducted between 9:00 AM and 1:00 PM and between 2:00 and 6:00 PM. During the exposure, the phone was switched on for 15 seconds every half an hour and it was controlled by automatic electronic system using fax signal. In the programmed time intervals, the connection was established for a definite time period. The mobile phone as a final terminal had a function of an automatic receipt of connection switched on. The connection was not cancelled, as it happens in case of lack of phone call, due to a fax signal, which was active during the whole time of the connection.

The mobile phone operated in a silent mode; it means that both sound of a bell and sound in the receiver were switched off, so the animals were exposed solely to electromagnetic field generated by the mobile phone.

The total number of connections for twenty-four hours was 16. The average density of the electromagnetic field recorded in the process of establishing the connection was *E*_1_ = 85.3 *μ*W/m^2^. The average density of the electromagnetic field throughout the established connection was *E*_2_ = 17.0 *μ*W/m^2^.

During the exposure, the animals were put in plastic cages. The mobile phone was placed directly under the cage in which the animals stayed during the exposure.

Measuring the density of electromagnetic field in an area dedicated to the exposure of rats to electromagnetic field was performed at fixed posts of the laboratory.

The laboratory workstation consisted of an electromagnetic field generator: Nokia multiband mobile terminal (far-field region of electromagnetic interaction). An outline of the laboratory workstation with five measuring points is demonstrated in Figure 1.

The average density of the electromagnetic field was calculated on the basis of sixteen consecutive measurements of the field's density, EMF [*μ*W/m^2^], recorded in five measuring points within the plastic cage by means of an electromagnetic interference meter TES-92.

The choice of only five measuring points was due to relatively large size of the meter's probe head.

### 2.3. Acquisition of Material for the Study of Skeletal System and for Biochemical Marking

After a time of adaptation to the place selected and adjusted to the electromagnetic field exposure (at the Faculty of Electrical Engineering, Silesian University of Technology, Gliwice, Poland), about 0.5 mL of blood was collected from the tails of rats in both control (*C*) and experimental group (*E*_*m*_). The procedure was repeated after 1, 2, 3, and 4 weeks. After centrifuging the clot of the collected blood (4 thousand revs./min, *t* = 10 min), serum was collected, which was then frozen in temperature of 253 K (−20°C). After finishing the experiment, serum samples were unfrozen. Next, the concentration of osteocalcin (OC), cross-linked N-terminal telopeptide of collagen alpha I chain (NTx), pyridinoline (PYD), calcium, and phosphorus in the serum was measured.

After 28 days of exposure to the electromagnetic field generated by a mobile phone (*E*_*m*_) or after 28 days of sham exposure to the field (*C*), the animals were anaesthetized with a mixture of xylazine (10 mg/kg* ip*) and ketamine (100 mg/kg* ip*). During deep sedation, about 2 mL of blood was collected from the rats' left heart ventricle. After centrifuging the clot of the collected blood (4 thousand revs./min, *t* = 10 min), serum was collected with the aim of measuring the concentration of osteogenesis and bone resorption markers, such as osteocalcin (OC), cross-linked N-terminal telopeptide of collagen alpha I chain (NTX), pyridinoline (PYD), calcium, and phosphorus. After collecting the blood, the rats, still anesthetized, were killed by dislocating the spinal cord. Later, selected bones were isolated: right femur, right tibia, and L_4_ vertebra.

### 2.4. Study of Bones' Macrometric Parameters

The isolated bones—right femur, right tibia, and L_4_ vertebra—were cleaned of soft tissue and weighed directly after cleaning on analytical scales with accuracy up to 0.0001 g. The mass of the isolated bones was expressed in mg and calculated in proportion to 1 kg of body weight (the mass of the animals' bodies was evaluated directly before applying general anaesthesia).

The length of the femur and tibia, as well as the diameters of diaphyses (both in coronal and in sagittal plane) and the diameters of both the proximal tibial epiphysis and the distal femoral epiphysis, was measured with an electronic calliper with accuracy up to 0.1 mm. The results of the measurements were expressed in millimeters [mm].

### 2.5. Study of the Bones' Mechanical Properties

The study of bones' mechanical properties of diaphysis and neck of the right femur and proximal right tibial metaphysis and diaphysis was performed with an INSTRON 3342 apparatus coupled with a computer equipped with Bluehill file 2.13 program.

The isolated femur or tibia was placed horizontally in the apparatus and either a linearly increasing pressure exerted in the middle of the diaphysis and perpendicularly to the axis of the femur or a pressure exerted in the area of the proximal tibial metaphysis (3 mm distal from the proximal articular surface) perpendicularly to the axis of the bone was applied. A three-point bend test was employed. The rate of rise of pressure force was 100 N/min.

On the basis of the analysis of the chart showing stress and strain of the plastic deformation in the diaphysis and in the right femoral neck and in a proximal right tibial metaphysis, the following parameters were determined: yield point force [N], yield point strain (translocation of external surface of pressured part of bone in relation to initial position) [mm], yield point energy [mJ], and yield point stress [MPa].

In the case of resilience of the right femoral epiphysis and the proximal right tibial metaphysis, the analysis of the results allowed determining the maximum bending force [N], strain at maximum bending force [mm], energy at maximum bending force [mJ], and stress at maximum bending force [MPa].

In the case of permanent deformation (fractures), the study allowed determining the following parameters for right femoral diaphysis and neck and proximal right tibial metaphysis: force at fracture [N], strain at fracture (spatial translocation of external surface of a pressured part of bone in relation to the position of the rest of the bone surface) [mm], energy at fracture [mJ], and stress at fracture [MPa].

### 2.6. Estimation of the Mineral Substance Mass in Bones

Right tibias (after having determined the mechanical properties) and L_4_ vertebrae were placed in porcelain crucibles. Next, bones were subjected to mineralization in muffle furnace at 640°C for 48 h in order to remove water and organic substances. Mineralized bones were weighed on analytical scales with accuracy up to 0.0001 g estimating the mineral substance mass. The determined mass of mineral substances was calculated in proportion to 100 g of bone mass determined directly after the isolation. The results of estimating the mass of mineral substances in bones were expressed in milligrams for 100 milligrams of bone mass [mg/100 mg].

### 2.7. Estimation of Calcium and Phosphorus Content in Bones

After mineralizing and estimating the mass of the mineral substance in tibias and L_4_ vertebrae, 100 mg of the mineral substances was weighed out and solved in 1 mL 6 M of hydrochloric acid solution for 24 h. The resulting solution was diluted with deionized water (560 times). Next, the content of calcium and phosphorus was determined by means of diagnostic sets: Calcium-MTB and Phosphorus (BioSystems).

### 2.8. Measuring the Concentration of Calcium and Phosphorus in Peripheral Blood Serum

Measuring the concentration of calcium and inorganic phosphorus was performed on blood serum collected from left heart ventricle immediately before killing the animals. A colorimetric method of measuring calcium and phosphorus was employed, using diagnostic tests: Calcium-MTB and Phosphorus (BioSystems). The results of measuring the calcium and phosphorus concentration were expressed in milligrams per 100 mL of serum [mg/dL] or in millimoles per liter [mmol/L] − calcium or nanomoles per liter [nmol/L] − phosphorus.

### 2.9. Measuring the Concentration of Osteogenesis Marker: Osteocalcin (OC) and Bone Resorption Markers—Cross-Linked N-Terminal Telopeptide of Collagen Alpha I Chain (NTX) and Pyridinoline (PYD) in Peripheral Blood Serum

Measuring the concentration of osteocalcin and cross-linked N-terminal telopeptide of collagen alpha I chain was performed on blood serum collected from rats' tails before beginning the exposure to electromagnetic fields and after 1, 3, and 4 weeks since the beginning of the exposure to electromagnetic field. Measuring the concentration of osteocalcin, cross-linked N-terminal telopeptide of collagen alpha I chain, and pyridinoline was performed additionally on blood serum collected from left ventricle directly before killing the animals. The concentration of the aforementioned markers of bone turnover in serum was measured using colorimetric immunoenzymatic method (ELISA), using the following tests: Rat-MID Osteocalcin EIA (Immunodiagnostic Systems), Osteomark NTx Serum ELISA (Osteomark), and MicroVue Serum PYD EIA Kit (Quidel).

### 2.10. Statistical Analysis

The results were statistically analyzed using the following computer programs: Statistica 7.1 PL, Statsoft, and Excel 2003, Microsoft. The statistical estimation was performed on the basis of analysis of variance (ANOVA), after prior checking of the homogeneity of variance by means of Levene's test and the normality of distribution in specific groups. If the homogeneity of variance (*P* > 0.05) was detected in Levine's test, single factor parametric ANOVA (Student *t*-test) was used. After finding statistically significant differences (nonhomogenous variance), Snedecor's *F* distribution was used. In each instance, a variance analysis of the results obtained in experimental (field exposure) group rats and in control group rats was performed. The assumed level of significance was *P* < 0.05.

## 3. Results

### 3.1. Macrometric Bone Parameters

Macrometric parameters of right femur and right tibia, such as mass, length, diameter of diaphysis, and diameter of distal and proximal epiphysis, were not significantly different among the rats exposed to the electromagnetic field generated by a mobile phone and in control group rats (Table 1).

No statistically significant differences between the results in control group rats and the results in experimental group rats were shown, using the ANOVA variance analysis.

The average mass of the L_4_ vertebra calculated in proportion to a kilogram of body weight in experimental group rats was 0.973 ± 0.009 mg/kg of body weight and it was statistically significantly lower by 12.5% (*P* = 0.0465) in comparison with the control group rats (1.112 ± 0.005 mg/kg of body weight) (Table 2).

### 3.2. Mechanical Properties of Bones

#### 3.2.1. Plastic Deformations

Four-week exposure of rats to the electromagnetic field generated by a mobile phone (Nokia 5110, 900 MHz) did not influence in a significant way the plastic deformation, including bending resistance of right femoral diaphysis and neck and proximal right tibial metaphysis, regarding such measured parameters as yield point force, yield point strain, yield point energy, yield point stress, yield point time, maximum bending force, strain at maximum bending force, and time at maximum bending force in comparison with the control group (Table 3).

Energy at maximum force bending of the proximal right tibial metaphysis was statistically significantly larger in rats from the experimental group (*E*_*m*_) than in rats from the control group (*C*) by 10.89% (*P* = 0.0446). Stress in proximal right tibial metaphysis at maximum bending force was statistically significantly lower in rats from the experimental group (*E*_*m*_) than in rats from control group (*C*) by 13.53% (*P* = 0.0443) (Table 3).

#### 3.2.2. Permanent Deformations (Fractures)

No significant influence of the electromagnetic field generated by a mobile phone (Nokia 5110, 900 MHz) on mechanical properties of the right femoral diaphysis and neck was shown regarding permanent deformation established by measuring parameters, such as force at fracture, strain at fracture, energy at fracture, stress at fracture, and time at fracture (Table 4).

In the case of permanent deformation, a statistically significant decrease of stress at fracture of the proximal right tibial metaphysis in comparison with the control group by 8.53% (*P* = 0.0463) was observed (Table 4).

### 3.3. Content of Mineral Substances in Bones

The mass of mineral substances in the right tibia of rats from the experimental group (*E*_*m*_) was approximately the same as in the rats from the control group (*C*). Calcium content in 100 g of mineral substances in the right tibia of electromagnetic field-exposed rats was statistically significantly lower by 12.44% (*P* = 0.0037) in comparison with the control group (Table 5).

In the rats from the experimental group (*E*_*m*_), there appeared a statistically significant drop by 13.95% (*P* = 0.0056) in the calcium/phosphorus ratio in the right tibia in relation to the control group (Table 5).

No statistically significant differences were shown using variance analysis ANOVA when comparing the content of mineral substances calcium and phosphorus in L_4_ vertebra in rats from the control group and in rats after the four-week exposure to the electromagnetic field (Table 5).

### 3.4. Concentration of Bone Turnover Markers in Rats' Blood Serum

#### 3.4.1. Osteocalcin: Osteogenesis Marker

The concentration of osteocalcin (OC) in blood serum collected from rats' tail vein on day zero, that is, 2 hours before commencing the experiment, in rats from the control group was 95.99 ng/100 mL, and it did not differ significantly from the concentration of OC in blood serum in rats from the electromagnetic field-exposed group. In rats from the control group, the concentration of this marker in blood serum was subject to gradual decrease during the next four weeks of the experiment (Table 6).

Four-week exposure of rats to the electromagnetic field generated by a mobile phone (*E*_*m*_) significantly increased the concentration of OC in the rats' blood serum after the first week of exposure by 27.39% (*P* = 0.001) in comparison with rats from the control group (*C*), whereas, in the other time periods (i.e., after the second, third, and fourth weeks of the exposure), the concentration of this marker was not significantly different from its concentration in blood serum of the rats from the control group. The increases observed, respectively, by 2.85%, 5.89%, and 5.94%, were not statistically significant (Table 6).

#### 3.4.2. Cross-Linked N-Terminal Telopeptide of Collagen Alpha I Chain (NTx): Bone Resorption Marker

In rats both from the control group and from the experimental group before the exposure to the electromagnetic field, the concentration of NTx in blood serum was similar and amounted on average to 3.55 nM BCE/L. In rats from the control group, the concentration of NTx in blood serum during the four weeks of the experiment was subject to gradual decrease from 3.55 to 2.72 nM BCE/L. A similar drop in the concentration of NTx in blood serum was observed also in rats from the experimental group: from 3.54 to 2.88 nM BCE/L, while the observed decreases of concentration of this marker in blood serum were smaller than in the rats from the control group. The differences in concentration of this marker observed in each time period were not statistically significant (Table 6).

#### 3.4.3. Pyridinoline: Bone Resorption Marker

In rats from the control group, the concentration of pyridinoline (PYD) in blood serum after four weeks of the experiment was 3.72 nmol/L, while, in the rats from the experimental group, after this time it was higher by 26.76% (*P* = 0.032). The observed increase was statistically significant (Table 6).

### 3.5. Concentration of Total Calcium and Total Phosphorus in Rats' Blood Serum

The four-week exposure of rats to the electromagnetic field generated by a mobile phone did not change the concentration of total calcium and the concentration of total phosphorus in those animals' blood serum in comparison with the control group (Table 7).

## 4. Discussion

The study of the influence of the electromagnetic field generated by a mobile phone on the functioning of the human skeletal system for experimental reasons solely would be against medical ethics. In order to thoroughly examine the effects of the influence of this field on the processes of bone remodelling, it was necessary to conduct this study using laboratory animals. Such research is usually performed on rats since these animals are characterized by fast bone turnover and their bone metabolism, similar to other mammals, proceeds in a manner similar to humans' [12, 13]. In accordance with commonly accepted norms, aiming at reducing the suffering of laboratory animals, the study was conducted on a small, yet representative, group of rats (20 specimens) divided into two subgroups of 10 specimens each.

Young rats (12-week-old male rats of Wistar stock) whose process of bone development was already completed were chosen for the experiment. This research enabled the examination of bone remodelling processes under the influence of the electromagnetic field generated by a mobile phone Nokia 5110 (900 MHz) in unoperated specimens, in which, as opposed to in the case of humans, it was possible to isolate specific elements of the skeletal system. The assessment of the influence of the studied field on the processes of bone tissue remodelling in rats was made possible among others by measuring macrometric parameters, such as mass, length, and bone diameter of specific bones, such as right femur, tibia, and L_4_ vertebra.

In order to obtain a broader picture of the changes transpiring in bone tissue under the influence of the electromagnetic field, the following research was conducted: determining the content of mineral substances, including calcium and phosphorus in the bone studied, and measuring the length of long bones and their diameter taken at their midlength, as well as the diameter of the epiphysis and the metaphysis. The macrometric measurements of right femur and right tibia performed in this research have shown that four-week exposure to the electromagnetic field generated by a mobile phone causes the decrease of bone mass in right femur by approximately seven percent in comparison with the control group. It does not, however, have impact on the mass of the right tibia, but it does reduce the bone mass of L_4_ vertebra.

Simultaneously, it was observed that exposure to the electromagnetic field generated by a mobile phone lowers the concentration of calcium in the mineral substance of tibia and it lowers the calcium/phosphorus ratio in 100 mg of this bone's mineral substance. The data obtained indicate that 4-week exposure to such electromagnetic field results in minor increase in the length of femur and tibia with an increase in diameter of the diaphysis and of proximal tibial epiphysis at the same time. The changes observed were not statistically significant.

Comparing the content of mineral substances in the rats from the control group with the rats exposed to the electromagnetic field generated by a mobile phone, it was possible to state that the influence of such electromagnetic field leads to a statistically significant decrease of calcium content and minor decrease in the mass of mineral substances in L_4_ vertebra.

So far, there has been little literature concerning the influence of the electromagnetic fields generated by a mobile phone on the macrometric parameters of long bones in rats. The results of this research can be a contribution to the study of the influence of the electromagnetic fields on the development of changes in the skeletal system on the basis of the evaluation of bone's micrometric parameters.

The analysis of available data suggests that the exposure to low-frequency (40–50 Hz) electromagnetic field disrupts the processes of bone remodelling in long bones (composed mostly of compact bone tissue), as well as in short bones, which are formed mostly from cancellous bone tissue (in L_4_ vertebrae and the epiphyses of long bones) [14–18].

The study of the influence of the electromagnetic radiation generated by a mobile phone operating at the frequencies of 900 MHz and 1800 MHz on the bone mineral density (BMD) of the iliac bone wing was conducted by Atay et al. [8]. This research was conducted on a sample of 150 people at the ages of 21–57, who, during the period of approximately 6.2 years, were exposed to the electromagnetic radiation generated by a mobile phone (14.7 hours/d). The research showed that, in those people, the BMD of iliac bone wing was statistically significantly lower on the side exposed to the field.

The influence of the electromagnetic radiation generated by a mobile phone on the bone mineral density of the proximal femoral epiphysis was examined also by Saravi. He found that, in people using a mobile phone for a year minimum, the bone mineral density measured at the level of the greater trochanter was statistically significantly lower in the right femur exposed directly to the electromagnetic field generated by a mobile phone in comparison with the opposite side (left femur—not exposed directly to the field) [9].

The studies of the influence of the electromagnetic radiation generated by a mobile phone were performed on animals as well. Yildiz et al. studied the influence of the electromagnetic field at the frequencies of 900 to 1800 MHz generated by a mobile phone (30 min/day for 28 days) on bone mineral density in rats. They established that a statistically significant decrease of BMD in proximal femoral epiphysis appears in animals exposed to the studied field [19]. Fragopoulou et al. studied the influence of the electromagnetic radiation generated by a mobile phone on the development of skull bones in mouse embryos [10]. Their research showed that, in the skull bones of mice exposed to the studied field, there appeared anomalies in soft tissue covering the skull and in skull bones after birth. Histological and histomorphometric studies showed the ossification of skull bones and chest ribs, as well as a displacement of Meckel's cartilage, which forms part of the mandible. Additionally, the research indicated a stimulating effect of the electromagnetic field generated by a mobile phone on the calcification of the skull.

The results obtained in this research have shown that the ratio of calcium to phosphorus in the mass of mineral substances was 2.15 in the rats from the control group, which indicated that the main mineral substance in these bones of the rats from the control group was hydroxyapatite, in which the calcium to phosphorus ratio is exactly 2.15.

In rats exposed to the electromagnetic field generated by mobile phone (Nokia 5110, 900 MHz), a decrease in this ratio (Ca/P) was observed in the bone studied. It dropped to 1.85 in tibia and 1.98 in L_4_ vertebra.

This data may suggest that lowering the calcium content in bones is due to its escape into blood or a remodelling of the hydroxyapatite structure under the influence of the electromagnetic field.

The latter of the aforementioned hypotheses is supported by the lack of the influence of the electromagnetic field generated by a mobile phone on the concentration of total calcium and total phosphorus in blood serum, which was established in this study.

The remodelling of the hydroxyapatite in bones is possible when changes in pH and changes in mechanical stress appear [20–22]. A more acidic environment causes the transformation of part of hydroxyapatite into a chemical of the following structure: Ca_3_(PO_4_)_2_·CaH (PO)_4_, where the ratio of calcium to phosphorus is approximately 0.2 and amounts to levels equivalent to the results obtained in this research for rats exposed to the electromagnetic field generated by a mobile phone (1.85 and 1.98). The explanation of the reason behind the loss of calcium in bones while being exposed to the electromagnetic field requires further research supporting the validity of the presented hypotheses.

The structural changes in bones of rats exposed to the influence of the electromagnetic field generated by mobile phone (Nokia 5110, 900 MHz) were reflected in the changes of the mechanical properties of the right femoral diaphysis and neck, as well as proximal right tibial metaphysis.

What has significance for biomechanics of bones are concepts such as stress (pressure) and deformation (strain).

Pressure is defined as force acting upon a unit area and is expressed in Pascals [1 Pa = 1 N/m^2^]. Strain is defined as the percentage change in length or relative deformation. It is possible to differentiate several types of pressure: compressive stress, tensile stress, and shearing stress.

What has been studied in this research are mechanical properties of femoral diaphysis (compact structure) and femoral neck (cancellous structure), as well as the mechanical properties of proximal tibial metaphysis (cancellous structure).

On the basis of the resistance measurement, it was demonstrated that the electromagnetic field generated by a mobile phone (Nokia 5110, 900 MHz) does not significantly affect the mechanical properties of the studied long bones regarding plastic deformations and it only increases the energy at maximum bending force and decreases stress in the proximal tibial epiphysis at maximum bending force.

Regarding permanent deformations, the electromagnetic field generated by a mobile phone causes characteristic decrease of stress at fracture in proximal tibial metaphysis.

Stress is a quantity that expresses the internal forces inside a body induced by external deforming force.

The decrease of stress in long bones under the influence of the electromagnetic field generated by a mobile phone observed in this study indicates that bones are less susceptible to the influence of external forces (pressure and stress) after being exposed to the field.

Due to the lack of sufficient data concerning the influence of electromagnetic fields on mechanical properties of mammals' bones, the changes in some parameters characterizing mechanical strength of bones observed in this study are insufficient to formulate a definite conclusion regarding the influence of the aforementioned fields on the biomechanics of bones and require more detailed research in this area.

The changes in bone remodelling in this study were evaluated on the basis of marking the concentration of the following markers of bone turnover in blood serum: osteocalcin (OC), osteogenesis marker, as well as cross-linked N-terminal telopeptide of collagen alpha I chain (NTx) and pyridinoline (PYD), bone resorption markers.

The obtained data regarding marking the concentration of osteocalcin in the serum of blood collected from tail vein every 7 days throughout the 28-day exposure to the field studied indicates that the level of this marker was subject to change in rats from the experimental group. Before the beginning of the exposure to the field, the concentration of osteocalcin in blood serum was comparable in both control group rats and experimental group rats. After one-week exposure to the field generated by a mobile phone, the concentration of osteocalcin was statistically significantly higher in experimental group rats than in control group rats, whereas, in the following weeks, it was not significantly different from the concentration in blood serum of control group rats.

Numerous in vitro and in vivo studies have conclusively shown that pulsed electromagnetic field stimulates maturation, differentiation, and activity of osteoblasts [6, 23–28]. Cheng et al. [25] demonstrated that sinusoidal pulsed electromagnetic field stimulates differentiation and maturation of rats' osteoblasts by means of activating NO-cGMP-PKG pathway. The study indicates that the effects of electromagnetic fields lead to increased activity of nitric oxide synthase (NOS), increased “Osterix” gene expression for transcription factor participating in the process of osteoblast differentiation, and increase in alkaline phosphatase activity and in the number of mineralized bone nodules.

The study of the influence of pulsed electromagnetic fields on bone tissue was carried out both in vitro and in vivo on humans.

Luo et al. studied the influence of pulsed electromagnetic fields with a field intensity of 1.1 mT and different frequencies applied for 30 minutes per day for 21 days on the differentiation of human mesenchymal stem cells [29]. In order to evaluate the level of mesenchymal stem cells osteoblast differentiation, both the activity of alkaline phosphatase and the level of osteocalcin gene expression were marked. The study revealed that the influence of the studied fields differs depending on the frequency; the highest level of osteoinduction was achieved in fields of 50 Hz frequency.

There exists no data in the literature regarding the influence of the electromagnetic field generated by a mobile phone (900 MHz) on the maturation, differentiation, and activity of the cells taking part in the bone remodelling processes (osteoblasts, osteoclasts, and osteocytes) and on the speed of bone turnover estimated by means of osteogenesis markers and bone resorption markers.

The data obtained in this study indicate that the electromagnetic field generated by a mobile phone (Nokia 5110, 900 MHz) stimulates the processes of osteogenesis in the first week of exposure, whereas, in the following weeks of exposure, it does not stimulate this process. Examining the specific mechanism of the fields' influence on the processes of bone remodelling requires further detailed study.

The speed of bone turnover is estimated by means of both osteogenesis markers and bone resorption markers [30]. One of the markers of bone resorption is cross-linked N-terminal telopeptide of collagen alpha I chain (NTx). It is released into blood during osteoclastic bone resorption and is created in the process of collagen degradation after removing N-terminal propeptides by specific enzymes (cathepsins, collagenases, and collagenolytic enzymes). Cross-linked N-terminal telopeptides are released into bloodstream and later excreted in urine in free forms (PYD or DPD) or in peptide-bound forms. NTx is a highly specific indicator of bone metabolism [30, 31].

The results of NTx marking in the blood serum of rats exposed to the electromagnetic field generated by Nokia 5110 (900 MHz) mobile phone have shown that the field does not have a significant influence on the processes of bone resorption in rats.

Pyridinoline (PYD), like NTx, is created in the process of collagen I degradation during osteoplastic bone resorption and is excreted with urine in free form, whereas NTx is excreted in peptide-bound form [30, 32]. The results of pyridinoline marking in blood serum after four weeks of exposure to the electromagnetic field generated by Nokia 5110 mobile phone obtained in this study have indicated an increased concentration of the marker in rats' blood serum. The observed significant increase of pyridinoline concentration in blood serum of the rats exposed to the electromagnetic field generated by a mobile phone in comparison with control group, where no increase of NTx concentration was observed, may be due to a higher specificity of NTx marking for bone metabolism in comparison with PYD or due to differences in sensitivity of the marking method of the markers (higher for NTx and lower for PYD)

Detailed analysis of the mechanisms of the influence of the electromagnetic field generated by a mobile phone on the processes of osteogenesis and bone resorption requires further in-depth study.

In spite of the growing prevalence of mobile phones in human environment, there is still little research regarding the influence of the electromagnetic fields generated by a mobile phone on the skeletal system of mammals.

The sparse evidence available seems to be indicating the negative biological effects of the influence of electromagnetic fields on living organisms (genotoxicity, carcinogenicity, thermal effects, and CNS function disorders). Research in this area is fragmentary and requires elaboration.

The results of this study do not confirm the negative influence of the electromagnetic field generated by Nokia 5110 (900 MHz) on the skeletal system (on the mechanical properties and bone turnover processes) of mammals.

## 5. Conclusion

The electromagnetic field generated by Nokia 5110 (900 MHz) mobile phone does not have a direct impact on the macrometric parameters of bones; however, it alters the processes of bone mineralization and the intensity of bone turnover processes (osteogenesis and bone resorption) and thus influences the mechanical strength of bones.

## Figures and Tables

**Table 1:** Macrometric parameters of femur and tibia.

Parameter measured	Bone studied			
	Femur		Tibia	
	Animal group		Animal group	
	*C* (*n* = 10)	*E*_*m*_ (*n* = 10)	*C* (*n* = 10)	*E*_*m*_ (*n* = 10)
Mass [mg/kg of body weight]	2.888 ± 0.016	2.710 ± 0.008	1.993 ± 0.006	1.993 ± 0.01
Length [mm]	35.35 ± 1.61	38.68 ± 1.64	38.32 ± 0.61	39.04 ± 0.64
Diameter of diaphysis [mm]	3.81 ± 0.15	3.89 ± 0.11	2.27 ± 0.12	2.43 ± 0.16
Diameter of the distal epiphysis [mm]	6.81 ± 0.17	6.81 ± 0.20	—	—
Diameter of the proximal epiphysis [mm]	—	—	7.08 ± 0.19	7.29 ± 0.24

The results are presented as arithmetic mean ± standard deviation (*x* ± *σ*_*n*−1_); *n*: number of rats in a group (male rats of Wistar strain, 10 weeks old, in the beginning of the experiment).

**Table 2:** Macrometric parameters of L_4_ vertebra.

Parameter measured	Bone studied	
	L_4_ vertebra	
	Animal group	Animal group
	*C* (*n* = 10)	*E*_*m*_ (*n* = 10)
Mass [mg/kg of body weight]	1.112 ± 0.06	0.973 ± 0.09

The results are presented as arithmetic mean ± standard deviation (*x* ±  *σ*_*n*−1_); *n*: number of rats in a group (male rats of Wistar strain, 10 weeks old, in the beginning of the experiment).

**Table 3:** Plastic deformation of bones.

Parameter measured	Bone studied					
	Femoral diaphysis		Femoral neck		Proximal tibial metaphysis	
	Animal group		Animal group		Animal group	
	*C* (*n* = 10)	*E*_*m*_ (*n* = 10)	*C* (*n* = 10)	*E*_*m*_ (*n* = 10)	*C* (*n* = 10)	*E*_*m*_ (*n* = 10)
Yield point force [N]	32.349 ± 4.703	34.828 ± 4.614	96.618 ± 9.221	95.840 ± 8.120	22.365 ± 2.001	24.028 ± 4.614
Yield point strain [mm]	0.615 ± 0.077	0.648 ± 0.087	0.832 ± 0.054	0.875 ± 0.095	0.315 ± 0.047	0.318 ± 0.087
Yield point energy [mJ]	5.254 ± 0.556	5.365 ± 0.596	38.03 ± 4.08	38.06 ± 4.20	7.354 ± 0.606	7.565 ± 0.786
Yield point stress [MPa]	19.895 ± 2.093	22.807 ± 2.031	—	—	15.295 ± 1.693	14.807 ± 2.431
Yield point time [s]	—	—	83.221 ± 5.458	85.111 ± 6.002	—	—
Maximum bending force [N]	46.841 ± 5.826	48.077 ± 4.169	98.107 ± 7.723	97.982 ± 8.477	36.541 ± 4.016	36.077 ± 4.169
Strain at maximum bending force [mm]	0.992 ± 0.163	1.020 ± 0.143	0.840 ± 0.042	0.849 ± 0.097	1.092 ± 0.173	1.020 ± 0.143
Energy at maximum bending force [mJ]	46.351 ± 5.309	43.325 ± 5.066	34.07 ± 5.10	37.02 ± 5.40	30.051 ± 4.309	**33.325 ± **5.066^*^ (*P* = 0.0446)
Stress at maximum bending force [MPa]	47.065 ± 3.578	5.926 ± 5.139	—	—	30.065 ± 3.578	**25.996 ± **2.139^*^ (*P* = 0.0443)
Time at maximum bending force [s]	—	—	84.032 ± 4.245	84.942 ± 8.677	—	—

The results are presented as arithmetic mean ± standard deviation (*x* ± *σ*_*n*−1_).

*n*: number of rats in a group (male rats of Wistar strain, 10 weeks old, in the beginning of the experiment).

^*^Statistically significant difference in relation to control group (dependent *t*-test).

**Table 4:** Permanent deformation of bones.

Parameter measured	Bone studied					
	Femoral diaphysis		Femoral neck		Proximal tibial metaphysis	
	Animal group		Animal group		Animal group	
	*C* (*n* = 10)	*E*_*m*_ (*n* = 10)	C (*n* = 10)	*E*_*m*_ (*n* = 10)	*C* (*n* = 10)	*E*_*m*_ (*n* = 10)
Force at fracture [N]	36.863 ± 3.275	37.210 ± 4.805	97.393 ± 7.647	97.138 ± 8.659	28.871 ± 3.115	27.970 ± 3.805
Strain at fracture [mm]	1.689 ± 0.033	1.674 ± 0.221	0.850 ± 0.045	0.858 ± 0.089	1.798 ± 0.033	1.742 ± 0.221
Energy at fracture [mJ]	64.481 ± 7.896	61.396 ± 7.428	36.11 ± 3.28	39.06 ± 4.23	54.481 ± 7.896	51.396 ± 7.428
Stress at fracture [MPa]	24.263 ± 2.168	23.019 ± 3.144	—	—	14.233 ± 2.168	**13.019 ± **3.144^*^ *P* = (0.0463)
Time at fracture [s]	—	—	85.035 ± 4.625	85.890 ± 8.577	—	—

The results are presented as arithmetic mean ± standard deviation (*x* ± *σ*_*n*−1_).

*n*: number of rats in a group (male rats of Wistar strain, 10 weeks old, in the beginning of the experiment).

^*^Statistically significant difference in relation to control group (dependent *t*-test).

**Table 5:** Content of mineral substances in bones.

Parameter measured	Bone studied			
	Tibia		L_4_ vertebra	
	Animal group		Animal group	
	*C* (*n* = 10)	*E*_*m*_ (*n* = 10)	*C* (*n* = 10)	*E*_*m*_ (*n* = 10)
Mass of mineral substances [mg/100 mg of bone mass]	40.43 ± 4.46	39.99 ± 3.16	32.34 ± 2.74	33.06 ± 3.16
Calcium content [mg/100 mg mineral substance]	41.63 ± 4.09	**36.45 ± **2.44^**^ (*P* = 0.0037)	38.63 ± 4.28	36.23 ± 1.94
Phosphorus content [mg/100 mg mineral substance]	18.74 ± 1.60	19.68 ± 1.05	18.21 ± 1.24	18.30 ± 1.52
Calcium/phosphorus ratio in 100 mg mineral substance	2.15 ± 0.22	**1.85 ± **0.11^*^ (*P* = 0.0056)	2.12 ± 0.19	1.98 ± 0.25

The results are presented as arithmetic mean ± standard deviation (*x* ± *σ*_*n*−1_).

*n*: number of rats in a group (male rats of Wistar strain, 10 weeks old, in the beginning of the experiment).

^*^Statistically significant difference in relation to control group (dependent *t*-test).

^**^Snedecor's *F* test.

**Table 6:** Concentration of bone turnover markers in rats' blood serum.

Time period of exposure to field (weeks)	Concentration of the measured bone turnover marker in blood serum					
	OC (bone GLA protein) [ng/100 mL]		NTx (collagen type I cross-linked N-telopeptide) [nM BCE/L]		PYD (pyridinoline) [nmol/L]	
	Animal group		Animal group		Animal group	
	*C* (*n* = 10)	*E*_*m*_ (*n* = 10)	*C* (*n* = 10)	*E*_*m*_ (*n* = 10)	*C* (*n* = 10)	*E*_*m*_ (*n* = 10)
0	95.99 ± 10.18	101.78 ± 11.24	3.55 ± 0.38	3.54 ± 0.39	—	—
1	56.04 ± 6.11	**71.39 ± **7.44^*^ (*P* = 0.001)	3.23 ± 0.48	3.25 ± 0.40	—	—
2	48.65 ± 5.002	50.04 ± 5.33	3.02 ± 0.26	3.05 ± 0.33	—	—
3	37.34 ± 4.11	39.54 ± 3.96	2.81 ± 0.29	3.05 ± 0.25	—	—
4	35.69 ± 3.39	37.81 ± 2.63	2.73 ± 0.31	2.88 ± 0.37	3.72 ± 0.30	**4.72 ± **0.54^**^ (*P* = 0.032)

The results are presented as arithmetic mean ± standard deviation (*x* ± *σ*_*n*−1_).

*n*: number of rats in a group (male rats of Wistar strain, 10 weeks old, in the beginning of the experiment).

^*^Statistically significant difference in relation to control group (dependent *t*-test).

^**^Snedecor's *F* test.

**Table 7:** Concentration of total calcium and total phosphorus in rats' blood serum.

Parameter in blood serum measured [nmol/L]	Animal group	
	*C* (*n* = 10)	*E*_*m*_ (*n* = 10)
Total calcium	2.59 ± 0.23	2.45 ± 0.25
Total phosphorus	1.04 ± 0.13	1.15 ± 0.15

The results are presented as arithmetic mean ± standard deviation (*x*  ±  *σ*_*n*−1_).

*n*: number of rats in a group (male rats of Wistar strain, 10 weeks old, in the beginning of the experiment).
